# Responding to Stress: Diversity and Resilience of Grapevine in a Changing Climate Under the Perspective of Omics Research

**DOI:** 10.3390/ijms26167877

**Published:** 2025-08-15

**Authors:** Tomas Konecny, Armine Asatryan, Hans Binder

**Affiliations:** 1 Interdisciplinary Centre for Bioinformatics, Leipzig University, 04107 Leipzig, Germany; tomas.konecny@abi.am; 2 Armenian Bioinformatics Institute, Yerevan 0014, Armenia; armine.asatryan@abi.am; 3 Institute of Molecular Biology of National Academy of Sciences, Yerevan 0014, Armenia

**Keywords:** viticulture, environmental stress, grapevine domestication and biodiversity, omics data, epigenetics

## Abstract

Climate change, with its altered precipitation and extreme temperatures, significantly threatens global viticulture by affecting grapevine growth, yield, and fruit quality. Understanding the molecular underpinnings of grapevine resilience is crucial for developing adaptive strategies. Our aim is to explore the application of multi-omics approaches (integrating genomics, transcriptomics, proteomics, metabolomics, and epigenetics) to investigate grapevine stress responses. Advances in these omics technologies have been pivotal in identifying key stress-response genes, metabolic pathways, and regulatory networks, particularly those contributing to grapevine tolerance to water deficiency, (such as drought and decreased precipitation), extreme temperatures, UV radiation, and salinity. Furthermore, the rich genetic reservoir within grapevines serves as a vital resource for enhancing stress tolerance. While adaptive strategies such as rootstock selection and precision irrigation are important, future research must prioritize integrated multi-omics studies, including those on regional climate adaptation and long-term breeding programs. Such efforts are essential to exploit genetic diversity and ensure the sustainability of viticulture in the evolving climate. In summary, this review demonstrates how utilizing the inherent genetic variability of grapevines and employing multi-omics approaches are critical for understanding and enhancing their resilience to the challenges posed by climate change.

## 1. Introduction

Grapevine (*Vitis vinifera* L.) has been cultivated for thousands of years, playing a crucial role in agriculture and the production of wine, one of the world’s most cherished beverages. However, the increasing unpredictability of climate change poses significant challenges to viticulture, necessitating a deeper understanding of the factors that contribute to grapevine resilience.

Climate change has introduced a range of stress conditions that can adversely affect grapevine growth, yield, and fruit quality. These stressors include severe weather events such as water deficiency and heatwaves. The impact of these changes is multifaceted, influencing not only the physiological processes of grapevines but also the timing of key phenological stages such as budbreak, flowering, and harvest. As a result, there is a growing need to explore both genetic and environmental factors that can enhance the resilience of grapevines to these stress conditions [1]. To achieve this, a comprehensive understanding of both genetic and environmental factors is required. This involves combining traditional breeding methods with modern genomic tools that can enhance the development of grapevines that are more resilient to stress conditions along with the implementation of adaptive management practices helping the wine industry against the challenges posed by a changing climate.

Modern grapevine breeding approaches leverage a combination of traditional techniques and cutting-edge technologies to enhance stress resilience and fruit quality [2]. In vitro culture techniques, for instance, allow for the assessment of ripening responses of different genotypes to environmental factors such as light and temperature, facilitating the selection of resilient grapevines. The advent of genome editing techniques such as CRISPR/Cas9 represents a paradigm shift in grapevine breeding [3]. These techniques offer precise modifications to grapevine genomes, enabling targeted improvements in stress resilience and fruit quality. Nevertheless, the practical implementation of CRISPR in perennial crops like grapevine is complex, as long generation cycles and regulatory hurdles slow down the adoption of these technologies in breeding programs [3]. Genomic-assisted breeding, on the other hand, integrates diverse omics datasets to identify genes underlying complex traits, optimizing the selection of parent lines and enhancing breeding efficiency [4]. The knowledge gained from omics studies has practical applications in grapevine breeding programs. For instance, polyploidization, combined with omics approaches, has been explored as a method to enhance genetic diversity and improve traits related to stress tolerance [5,6]. Omics refers to the large-scale study of biological molecules, encompassing fields such as genomics (the analysis of entire genomes), transcriptomics (the study of RNA transcripts), proteomics (the examination of proteins), and metabolomics (the analysis of metabolites). Such comprehensive approaches further our understanding of gene expression changes under combined abiotic stress [7,8,9]. Their integration provides a robust framework for elucidating complex gene regulatory networks and offers molecular targets for enhancing stress resilience in grapevine breeding programs, an effort that is increasingly crucial in the context of climate change. The ongoing development of omics technologies holds potential to enhance viticulture practices and support more sustainable vine and wine production, though long-term validation in practical settings remains essential.

A growing body of evidence highlights, for example, the central role of transcriptomics gene regulation in orchestrating grapevine and other fruit crop responses to abiotic stresses such as drought, salinity, and extreme temperatures. In grapevine, Transcription Factor (TF) families including MYB and WRKY have been shown to regulate stress-induced metabolic and signaling pathways. For example, *VhMYB2* positively regulates salt tolerance mechanisms by modulating antioxidant enzyme activity and ion homeostasis [10], while *VhWRKY44* enhances drought tolerance through activation of stress-responsive genes [11]. Similarly, *VhMYB15* has been linked to improved cold and salt tolerance by influencing proline accumulation and Reactive Oxygen Species (ROS) detoxification [12]. Beyond grapevine, other fruit crops offer important parallels. For instance, in *Malus baccata*, *MbMYBC1* enhances tolerance to both drought and salinity via regulation of abscisic acid (ABA)-dependent signaling pathways [13]. In *Fragaria vesca*, genes *FvMYB44* and *FvMYB114* have been reported to enhance drought and salt tolerance through transcriptional activation of stress-defense genes [14,15]. These studies underscore the conservation of transcriptional regulatory mechanisms across fruit crops, including grapevine.

**Figure 1:**
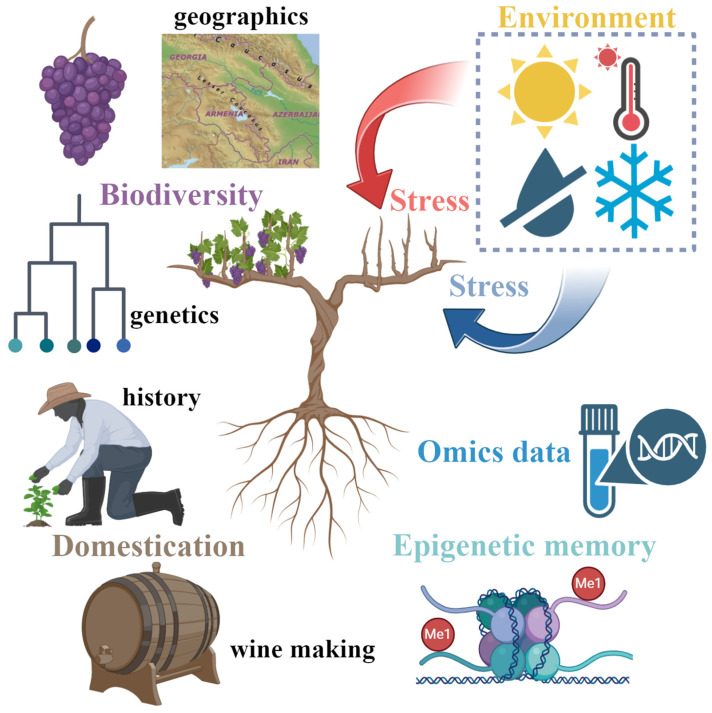
**Navigating stress factors of grapevine resilience, a schematic overview**. Biodiversity, domestication, environmental stress, and epigenetic memory collectively shape grapevine adaptation, while omics data reveal the intricate biological processes that determine its resilience to unfavorable conditions.

This review provides an overview of the genetic and environmental factors influencing grapevine resilience (Figure 1). In the first and second sections, we address the genetic diversity and evolutionary history of grapevines as key elements shaping their genetic architecture, with direct implications for stress resilience. Then, we explore the impact of abiotic stressors connected with climate change, such as water deficiency and extreme temperatures, on grapevine resilience. Afterwards, our focus shifts to omics technologies, including genomics and proteomics, which offer insights into the genetic mechanisms underlying stress tolerance. Finally, we delve into epigenetic regulation, emphasizing the role of epigenetics in the face of climate change. We conclude our review by highlighting recent research insights and their implications for viticulture.

## 2. Grapevine Evolution, Domestication, and Diversity

### 2.1. Responses to Climate and Environmental Changes Across the Millennia

While direct instrumental climate data for millennia are not available, paleobotanical and archaeological evidence serves as crucial proxy data, demonstrating the long-term interaction between environmental shifts and grapevine development. Early domestication of *V. vinifera* began in Western Asia and the Caucasus [16]. Grapevine cultivation later spread from the Near East to the Mediterranean Basin and beyond, becoming essential to various cultures and economies [17]. Today, *V. vinifera* remains the primary species used in the global wine and table grape production, covering 7.2 million hectares and yielding over 74 million tons of grapevines in 2023 (https://www.oiv.int/ (accessed on 6 August 2025)). Its adaptability to different climates and soil types allows it to flourish in diverse regions from sun-drenched Mediterranean vineyards to the cooler climates of Northern Europe and North America.

Guzmán-Ardiles et al. [18] emphasized the grapevine’s rich evolutionary history shaped by natural processes and human intervention in response to shifting climates and environmental pressures. Fossil records reveal that wild grapevines existed in the Northern Hemisphere during the Neogene and Paleogene periods (approximately 2.58 to 66 million years ago) [18]. Although the precise origins of grapevine domestication remain unclear, evidence indicates that it began around 9000 BC in regions such as the South Caucasus (modern-day Georgia and Armenia) and parts of Western Asia [19]. This period, following the end of the Wurmian glaciation, was marked by significant temperature fluctuations [20]. The melting of large ice caps led to massive freshwater influxes into the Atlantic Ocean, which is believed to have disrupted oceanic currents like the Gulf Stream, causing a temporary “rebound” in glaciation and colder, rainier conditions in regions such as the Black Sea coast, Anatolia, and Iran. These fluctuating environmental conditions influenced both human migration patterns and the opportunities for grapevine to differentiate and eventually be domesticated in separate geographic areas.

Archaeological findings, including ancient DNA analysis of Neolithic grape seeds, points to multiple domestication events and gene flow between wild and cultivated populations, complicating the linear “origin story” often presented [21,22]. Environmental factors, like varying temperatures, rainfall patterns, and soils, played a crucial role, as grapevines must have adapted when spreading across Europe and Asia. This adaptation was evolving through both natural selection and human-driven breeding [23,24]. For instance, the Areni-1 winery in Armenia (circa 4000 BC) [25] and findings from Georgia’s Gadachrili Gora region (dating back to 6000 BC) [26] underscore the long-standing link between grapevine cultivation and climate adaptation.

During the medieval period, European monastic networks were instrumental in preserving and diversifying grape varieties. Recent whole-genome sequencing studies indicate that these networks created genetic “hotspots,” enhancing resilience to environmental changes long before modern breeding techniques emerged [27]. These selection practices ultimately gave rise to grapevines suited for wine, table consumption, and raisin production. The environmental impact on grapevine evolution became particularly evident during the 19th century, when the introduction of North American grapevine species to Europe inadvertently brought pests like *Phylloxera*, devastating European vineyards [28]. In response to this ecological crisis, hybrid grapevines arose, combining *V. vinifera* with American species like *V. labrusca* or *V. riparia* to enhance pest tolerance [29,30]. Though initially controversial due to their distinct “foxy” flavors, these hybrids demonstrated remarkable adaptability to harsher climates and reduced the need for chemical treatments.

Today, renewed interest in hybrid grapevines like ‘Baco Noir’, ‘Seyval Blanc’, and ‘Clinton’ reflects a growing commitment to sustainable viticulture, supported by modern genetic tools like CRISPR that help reintroduce pest tolerance while preserving traditional wine profiles [31,32]. Future research is needed to determine if hybrid grapevines might outperform classic varieties in regions facing water deficiency and extreme temperatures [18,33].

**Figure 2:**
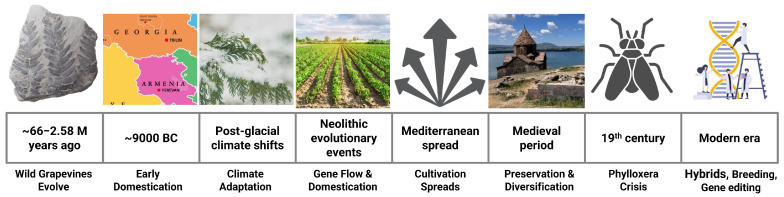
**Grapevine evolution, domestication, and diversity.** The timeline illustrates the evolutionary origin of wild grapevines during the Neogene and Paleogene periods (~66–2.58 million years ago) as revealed by fossil evidence. Early domestication of *Vitis vinifera* began around 9000 BC in Western Asia and the South Caucasus, coinciding with significant post-glacial climate fluctuations that influenced grapevine adaptation and human migration. Multiple domestication events and gene flow occurred during the Neolithic, facilitating the spread of cultivation into the Mediterranean Basin and beyond. During the medieval period, European monastic networks preserved and diversified grapevine varieties, enhancing resilience to environmental changes. The 19th-century Phylloxera infestation led to hybridization with North American species, resulting in pest-resistant hybrids. Recent advances in genetic tools such as CRISPR have renewed interest in hybrids to promote sustainable viticulture and improve stress tolerance under climate change.

Overall, exploring the domestication and evolution of grapevines is essential for enhancing their stress resilience in modern agriculture (Figure 2). By studying how these plants adapted over millennia, researchers and farmers can develop varieties that withstand challenges driven by the shifts in climate.

### 2.2. Genetic Diversity of Grapevines—A Reservoir for Stress Tolerance

The *Vitis* genus comprises both wild and cultivated grapevine species, with over 60 wild species distributed across North America (e.g., *V. riparia*, *V. rupestris*, *V. berlandieri*, *V. aestivalis*, *V. labrusca*), Asia (e.g., *V. amurensis*, *V. davidii*, *V. piasezkii*), and Europe (*V. sylvestris*), and several hundred wild cultivars in the Caucasus region [34,35]. Additional data from other regions of the post-Soviet space (particularly the North Caucasus, Crimea, southern Russia, and the Russian Far East) further enriches our understanding of Eurasian grapevine diversity. In Crimea, southern Russia, and Ukraine, studies have characterized regional cultivars such as ‘Kokur Belyi’ and ‘Krasnostop Zolotovskiy’ using Single-Nucleotide Polymorphism (SNP) profiling, confirming their distinctiveness and their value as local genetic resources [36]. Furthermore, wild grapevines (*V. vinifera* subsp. *sylvestris*) sampled from nature reserves such as the Utrish and Sudak regions of Crimea revealed significant genetic isolation from cultivated varieties, suggesting relic populations of wild origin [37].

The wild species harbor a wealth of genetic diversity, which has played a crucial role in the domestication and evolution of modern grapevines [17,21]. Many wild species demonstrate resilience to abiotic stresses, such as water deficiency, cold, and high soil pH, making them invaluable as rootstocks in vineyards and enhancing sustainability by conferring stress tolerance [38].

Domestication of grapevines is a complex process shaped by both natural and human-driven selection. Early domestication likely involved the selection of wild *V. sylvestris* plants that exhibited favorable traits such as larger berries, higher sugar content, and robust growth under cultivation. Over time, continued hybridization between cultivated *V. vinifera* and local wild species led to genetic exchange, enhancing diversity and adaptability [23]. For example, North American species like *V. riparia* contributed tolerance to phylloxera, a devastating pest, through hybridization events, which subsequently shaped breeding programs and vineyard management practices [39]. Additionally, the primary reservoirs of grapevine diversity are located in the Caucasus and the Middle East, where the presence of wild relatives and long-standing traditional cultivation under diverse environmental conditions has maintained a broad and valuable genetic base.

Whole-genome sequencing and genetic marker studies shed light on this intricate domestication history. Research involving 472 *Vitis* accessions revealed that cultivated grapevines retained signatures of gene flow from wild relatives, suggesting that hybridization played a continuous role throughout the domestication process [21]. This genetic exchange not only contributed to phenotypic diversity but also enhanced resilience by incorporating alleles associated with stress tolerance.

Despite these advancements, key knowledge gaps remain [40]. While genomic studies have illuminated broad patterns of diversity and domestication, they often overlook region-specific adaptations and the contributions of lesser-known wild species. Additionally, the focus has largely been on cultivated grapevines, leaving the full potential of wild *Vitis* species underexplored. Further investigation into the adaptive mechanisms of wild grapevines could uncover new genetic resources for breeding climate-resilient grapevines.

Understanding the domestication and dissemination history of grapevines offers crucial insights for modern breeding. It highlights the importance of preserving genetic diversity and leveraging wild species in breeding programs to enhance stress resilience and fruit quality. Future research should prioritize integrating diverse *Vitis* species into genomic studies, involving phylogenetic studies, to identify underutilized genetic resources, refine breeding strategies, and develop grapevines better suited to the challenges posed by climate change.

### 2.3. Diversity Linked with Domestication Journey

The domestication journey of the grapevine has intricately shaped its genetic diversity. While the initial phase of domestication from its wild progenitor, *Vitis vinifera* subsp. *sylvestris*, likely involved a reduction in genetic diversity due to a population bottleneck [35,41], cultivated grapevines today can exhibit surprisingly higher, and in some aspects comparable or even richer, diversity than their wild relatives. This phenomenon is largely attributed to subsequent millennia of human-driven evolutionary processes. Continuous and strong human selection for a multitude of desirable traits (such as berry size, color, sugar content, flavor profiles, and harvest time) has actively maintained and promoted genetic variants underpinning this phenotypic variation [42]. Consequently, these cumulative processes have generated the extensive catalogue of cultivated grapevine varieties seen today, each with a unique genetic makeup and often a complex mosaic of ancestries, contributing to a broad spectrum of diversity for important traits.

Domesticated grapevines typically exhibit highly heterozygous genomes, complicating genome assembly and analyses [43]. For example, the genome of ‘Chardonnay’ revealed that around 15% of its genes are hemizygous, containing many repetitive elements. To address this complexity, the highly homozygous PN40024 genotype, derived from the self-pollination of ‘Helfensteiner’, was selected for genome sequencing [44]. In terms of chromosomal configuration, wild and domesticated *Vitis* species generally have a diploid chromosome number of 2n = 38, representing 19 chromosome pairs in the subgenus *Euvitis*. Yet, species in the subgenus *Muscadinia*, such as *V. rotundifolia* and *V. munsoniana*, possess a chromosome number of 2n = 40 [40]. *Muscadinia* grapevines, which are native to North America, exhibit tolerance to pathogens and unique traits like late flowering and larger fruit sets. Despite these advantages, studies have shown that domesticated *Muscadinia* grapevines have experienced a reduction in genetic diversity, differentiating them from their wild relatives [40].

Recent advances in sequencing technology have enabled the assembly of genomes for multiple grapevines, like ‘Black Corinth’, ‘Cabernet Franc’, ‘Cabernet Sauvignon’, ‘Carménère’, ‘Chardonnay’, ‘Merlot’, and ‘Nebbiolo’ [45]. Nevertheless, many of these genome assemblies are incomplete, particularly in repetitive regions, highlighting the need for additional reference genomes better representing the genetic diversity of grapevines.

Structural variations and other genetic differences are associated with important traits in grapevines. For instance, the genome of *V. vinifera* cv. ‘Shiraz’ revealed a unique combination of *VviTPS24* variants linked to rotundone production and aromatic characteristics [46]. Moreover, *V. adenoclada*, a wild grapevine species with tolerance to heat and water deficiency, was studied through a multi-omics approach to identify beneficial genes for future breeding programs [47]. These findings emphasize the importance of utilizing the genetic diversity of wild and cultivated grapevines to enhance stress tolerance and adaptability.

### 2.4. Novel Perspectives: Caucasian Grapevines—Diverse but (Still) Understudied

In Central Europe and the western Balkan Peninsula, studies comparing wild grapevine populations (*V. vinifera* subsp. *sylvestris*) to cultivated grapevines (*V. vinifera* subsp. *vinifera*) found lower genetic diversity in wild grapevines. Interestingly, gene flow analyses indicated interactions between wild and cultivated grapevines, with cultivated grapevines from Central Europe showing greater genetic affinity to wild grapevines than those from the Balkan region [48]. This region emerged as a genetic hotspot, where wild and cultivated grapevines influence each other’s genetic diversity.

Different regions have cultivated unique grapevines, reflecting local adaptation and diversity. In Italy, studies of traditionally cultivated intra-varietal grapevines have revealed somatic variation within a single variety, as well as significant genetic divergence among grapevines from different habitats or with distinct origins [39]. The study identified divergent SNP loci linked to traits critical for grapevine phenology and environmental adaptation.

The importance of wild grapevines in breeding programs cannot be overstated. In the United States, muscadine grapevines serve as a valuable genetic resource, while in Europe, the conservation of wild grapevine populations faces significant challenges due to habitat loss and genetic erosion. Ongoing genetic diversity studies in the Caucasus and Central Asia regions have further identified these areas as crucial reservoirs of genetic variation, contributing to the shaping of modern grapevines [17].

A region of traditional wine making, the Caucasus is incredibly rich in diverse, but still understudied, grapevine accessions. Georgia, nestled in the heart of the Caucasus region, is known as one of the world’s oldest wine-producing countries, with a rich history of viticulture that dates back to 9000 BC [49]. Complementary archaeological investigations in Georgia documented a long history of viticulture through multidisciplinary approaches (including archaeological excavations, radiocarbon dating, and morphometric analyses of ancient grape seeds) which collectively illuminate the early phases of grapevine domestication in the region [49]. Furthermore, geometric morphometric analyses combined with ancient DNA extraction from grape pips traced the emergence of domesticated morphotypes to around 1000 BC, thereby linking modern grapevine diversity with its ancient progenitors [50].

Beyond the historical significance, the extensive genetic diversity of grapevines in Georgia is well documented. A comparative study of wild versus domesticated grapevine traits revealed that *V. vinifera* subsp. *sylvestris* exhibits faster fermentation rates despite higher phenolic contents, indicating untapped potential for improving wine quality and aging characteristics in modern winemaking practices [51]. A comprehensive review of Georgian grapevines demonstrated that these traces possess distinctive phenological and stress-tolerance features that may prove critical for adapting viticulture to future climate challenges [52]. In parallel, high-throughput SNP array analyses of Georgian genetic resources confirmed that the genetic variability within these grapevines is not only high but also distinct from that observed in European grapevines, supporting their potential for future breeding programs [53]. The convergence of data from ancient seed morphometrics, molecular genetics, and modern phenotyping reinforces the hypothesis that traditional grapevine cultivation practices in Georgia contributed significantly to the selection and propagation of desirable traits.

Recent studies revealed that Armenian wild grapevines represent an invaluable reservoir of Caucasian genetic diversity with significant potential for disease tolerance and crop improvement. Margaryan et al. [34] revealed how DNA-based marker analyses of wild *V. sylvestris* populations from various Armenian regions uncovered high allelic diversity and the presence of tolerance alleles against powdery mildew. Similarly, Dallakyan et al. [54] reinforce these findings by demonstrating extensive genetic variation among wild grapevine populations in the Caucasus and Near East, which underscores the evolutionary importance of these gene pools. In addition, the work of Margaryan et al. [55] on molecular fingerprinting and phylogenetic relationships among wild and cultivated grapevines in Armenia provides compelling evidence for gene flow between these groups, suggesting that indigenous grapevines may have partly inherited beneficial traits from their wild counterparts. First studies of the whole genome diversity of Armenian grapevine and of its relation to European and worldwide accessions using machine learning (using Self-Organizing Maps) are available [56,57,58].

Wild and early-domesticated grapevines are key sources of beneficial traits, including broad climate adaptability and tolerance to pests and diseases. Wild species in various regions show strong tolerance to environmental stress and pathogens. These traits are valuable for modern viticulture, especially in the face of climate change.

## 3. Environmental Factors Causing Abiotic Stress in Grapevine

Climate change introduces a range of abiotic stressors that can adversely affect grapevine growth, yield, and fruit quality. These weather changes reshape vineyard regions worldwide. For instance, Southern European regions, such as southern France and Spain, are experiencing increased heat stress and desertification, challenging grapevine quality and yields [59,60]. Meanwhile, cooler northern regions, including southern Sweden and parts of the UK, are becoming more suitable for viticulture, benefiting from longer growing seasons. Similar trends are seen in North America, where warming allows vineyards to expand further north into Canada while threatening established wine regions like California with heatwaves and wildfires [61]. As a result, one of the primary effects of climate change on grapevines is the compression of phenological stages, leading to earlier budbreak, flowering, and harvest dates [62]. This mismatch between the grapevine’s developmental stages and the optimal environmental conditions can reduce both yield and quality.

### 3.1. Heat and Drought

Extreme heat can negatively affect grapevine quality by accelerating sugar accumulation, disrupting acidity balance, and altering phenolic development [63]. Excessive water loss caused by increased evapotranspiration contributes to a reduction in grapevine vigor and alters berry composition. Water stress can lead to the accumulation of stress-related plant hormones such as ABA, which is critical for mediating water deficiency responses by inducing stomatal closure and activating drought-responsive genes [64]. In parallel, plant hormone ethylene plays a dual role by mediating heat stress responses (affecting berry ripening, cell wall modifications, and antioxidant systems) and modulating fruit ripening processes [65]. Additionally, while jasmonic acid is primarily recognized for its role in biotic stress responses, it is also involved in abiotic stress responses, often through complex hormonal crosstalk with abscisic acid, ethylene, and salicylic acid, though its function in abiotic stress can be context-dependent [66]. Heat stress also reduces photosynthesis efficiency and increases respiration, resulting in significant yield losses [67,68]. In grapevines, despite heat reducing malic acid content during maturation reducing berry quality, it increases sugar accumulation and flavonoid levels [63,69]. Additionally, the aroma and color of grape berries are decreased by lipids replaced by starch in leaf chloroplasts and other heat-induced changes, involving osmolyte production and secondary messengers like calcium ions, MAP kinases, and ROS [64,70,71]. Declining precipitation and prolonged water deficiency periods exacerbate water scarcity, particularly in Mediterranean wine regions. Water deficiency reduces berry size, affecting yield and altering tannin and anthocyanin concentrations, which are critical for wine quality. Research also suggests that grapevines under such water-deficit stress exhibit modifications in secondary metabolism, with increased flavonoid accumulation as a defense mechanism [72].

### 3.2. Cold and Freezing

Low temperature can be stratified into chilling (0 to 15 °C) and freezing (below 0 °C). Chilling stress disrupts photosynthesis, arrests enzymatic activities, and alters membrane lipids [70]. Freezing stress leads to ice crystal formation in the apoplast, causing osmotic stress and disrupting lipid polymorphism, which can damage cell membranes and lead to cell death. Plants deploy specific mechanisms, including the ICE1-CBF-COR transcriptional cascade (Inducer of CBF Expression 1–C-repeat Binding Factor–Cold-Responsive genes) and calcium signaling, to counteract cold stress [73,74]. Notably, grapevine species such as *V. amurensis* and *V. riparia*, which exhibit natural cold tolerance, serve as vital genetic resources for breeding programs aimed at improving cold tolerance [75,76]. Integrating omics approaches with breeding strategies is crucial for developing climate-smart grapevines capable of maintaining productivity and quality under temperature extremes [72,77]. Additionally, spring frosts, which occur after budbreak due to warmer winters, can damage tender tissues, necessitating adaptive measures such as late-budding grapevines bred from cold-hardy *Vitis* species [78,79,80].

### 3.3. UV Radiation and Soil Salinity

Beyond temperature extremes, high UV-B radiation induces oxidative stress by generating ROS, impairing photosynthesis and DNA integrity [81]. In response, grapevines synthesize UV-absorbing flavonoids and anthocyanins, thereby enhancing both UV shielding and antioxidant defenses. Similarly, soil salinity, intensified by irrigation with brackish water, disrupts ion homeostasis, causing osmotic stress [82]. To mitigate these effects, tolerance strategies include abscisic acid-mediated stomatal regulation and the use of rootstocks such as *V. berlandieri* × *V. rupestris* hybrids, improving salt tolerance and root vigor [83].

We summarized the impact of the various environmental stressors on grapevines, detailing affected regions, severity, morphological and biochemical changes, molecular mechanisms, and key genes involved in stress responses (Table 1).

## 4. Omics Strategies for Abiotic Stress Resilience

### 4.1. Genetic Insights into Grapevine Stress Tolerance

While contemporary *V. vinifera* cultivars exhibit relatively limited genetic diversity due to centuries of selective breeding, substantial variability persists in wild *Vitis* species and traditional landraces. This genetic richness underlies a broad range of adaptive traits that contribute to resilience against environmental stresses. Understanding the genetic basis of such traits is essential for developing improved grapevines with enhanced stress tolerance. The effort to unravel these genetic mechanisms began with traditional breeding approaches, which relied on phenotypic selection for characteristics such as drought tolerance and disease tolerance.

Dai et al. [104] developed a two-step in vitro culture system that combines fruiting-cuttings with organ in vitro culture, enabling long-term cultivation of grape berries through the full ripening process. While this system offers valuable insights into environmental influences on berry development and composition, its broader application to breeding programs requires further optimization to more accurately replicate vineyard conditions. Additionally, the in vitro culture system is used for intact detached grape berries, which actively absorb and utilize nutrients from the culture medium, exhibiting ripening features such as color change and softening. Yet, the extent to which these findings translate to field conditions remains a challenge [2].

Nevertheless, the existence of a broad reservoir of genetic diversity in wild and traditional grapevine populations contrasts with the limited diversity observed in the small number of cultivars dominating modern viticulture. The limited genetic diversity of *V. vinifera* cultivars and the slow pace of conventional breeding spurred the adoption of molecular tools in the late 20th century. Early efforts focused on genetic mapping of qualitative traits (e.g., disease tolerance loci like *Run1* and *Rpv1*), but these approaches struggled to dissect complex polygenic traits such as abiotic stress tolerance [105,106]. The completion of the first grapevine reference genome (PN40024) in 2007 marked a turning point, enabling genome-wide studies of stress adaptation [107]. This milestone catalyzed the shift from candidate gene approaches to systems-level omics strategies, including transcriptomics, proteomics, metabolomics. and recently, the application of machine learning in omics data analysis [108]. For instance, early transcriptomic studies in the 2010s revealed broad stress-responsive pathways, such as the abscisic acid signaling pathway and system of scavenging ROS [109,110].

### 4.2. Genome-Wide Association Studies and Beyond

The advent of next-generation sequencing and pangenomics helped to reduce this gap by capturing structural variations across wild and cultivated grapevine species [111,112]. Hereby, Genome-Wide Association Studies (GWASs) have revolutionized genetic research by enabling the identification of loci associated with complex traits across species. By analyzing genetic markers and their correlations with phenotypic traits, a GWAS facilitates the understanding of genetic architectures, particularly for quantitative traits influenced by multiple genes and environmental factors.

In *V. vinifera*, a GWAS dissects traits like berry quality, disease tolerance, and abiotic stress tolerance. Flutre et al. [113] identified multiple quantitative trait loci for various phenotypic traits, offering insights into their genetic underpinnings. The study highlights GWASs as a robust tool for elucidating grapevine genetic diversity and domestication patterns. GWASs also uncovered genes associated with stress resilience in grapevines. For example, strigolactone biosynthetic pathway genes were identified as critical for salt and water deficiency tolerance [114]. Similarly, autophagy-related genes were linked to tolerance against copper stress, a significant factor in vineyards due to fungicide use [115].

Beyond grapevines, GWASs identified stress-responsive genes in crops like rice and wheat with possible impacts for other plants including grapevine. In rice, *OsWRKY53* regulates salt tolerance [116], while in wheat, GWAS uncovered genomic regions contributing to water deficiency tolerance [117]. A recent comprehensive review of association mapping in grapevine further emphasizes the GWAS’s value in dissecting agronomic traits such as fruit quality, yield, and stress tolerance, while also discussing its future prospects in viticulture research [118]. These studies demonstrate the GWAS’s broad applicability in improving crop resilience. Grapevines pose unique challenges for GWASs due to their high heterozygosity and complex linkage disequilibrium. Still, advancements like the development of a grapevine pangenome enhance GWAS accuracy by accounting for structural variations and rare alleles [111].

Integrating GWASs with transcriptomic, physiological, and biochemical resources can provide more comprehensive insights into stress responses [119]. For example, Marrano et al. [120] conducted single-nucleotide polymorphism analysis comparing wild and cultivated grapevines and identified numerous “signatures of selection” linked to stress response and hormone signaling pathways, thereby highlighting the genetic basis for differential adaptation to environment. Additionally, Coupel-Ledru and her team identified six heat tolerance loci, named BLAZE, each containing single-nucleotide polymorphisms that explained up to 22% of genotypic variation in leaf firing magnitude, with BLAZE5.1 being significant across all traits [121]. Complementing these findings, the first high-quality draft sequence of the grapevine genome demonstrated that the grapevine genome is a composite of three ancestral genomes resulting from ancient hexaploidization events in angiosperms, and that no recent genome duplication has occurred [107]. Nowadays, the GWAS uses high-density SNP arrays and pangenomes to identify loci governing quantitative traits. For example, a GWAS identified β-amylase genes linked to cold tolerance [122].

The application of gene-editing technologies such as Clustered Regularly Interspaced Short Palindromic Repeats (CRISPR) and CRISPR-associated protein 9 (Cas9) (reviewed in [8]) could play a crucial role in enhancing the stress resilience of grapevine. These genomic tools hold promise for the creation of grapevines with improved resilience, fruit quality, and adaptive capacity under changing climatic conditions. Recent advancements in genomics have provided valuable insights into the genetic basis of stress tolerance, and the integration of these tools with traditional breeding techniques holds great promise for the future of grapevine research. This includes the use of CRISPR/Cas9 gene-editing technology precisely modifying key genes associated with grapevine resilience to stress [123]. This method utilizes the CRISPR-associated protein along with guide RNA to target specific genomic sequences, allowing for targeted gene disruption or insertion.

### 4.3. Genomic and Transcriptomic Studies

Genomics approaches, including reference genomes and genetic mapping, provide insights into the structure, function, and evolution of grapevine genomes, enabling the identification of genes associated with stress tolerance.

Marker-assisted selection and genomic selection are widely recognized as effective approaches for accelerating the development of stress-tolerant grapevines. Marker-assisted selection uses molecular markers linked to quantitative trait loci associated with desirable phenotypes (such as water-deficit and heat tolerance or disease tolerance) to facilitate early selection in breeding programs [124,125]. By contrast, genomic selection uses genome-wide, high-density markers to predict the genetic potential of individuals through statistical models trained on phenotypic and genotypic information from a reference population [126]. Genomic studies identified key transcription factors that regulate the expression of defense-related genes. For instance, the *MYB, WRKY, NAC,* and *bHLH* families of transcription factors are upregulated in response to abiotic stress, highlighting their role in the activation of stress-responsive pathways [127,128]. While marker-assisted selection has accelerated breeding efforts by identifying genetic variations linked to stress tolerance [129], genomic selection offers a more holistic approach, particularly for complex traits influenced by many small-effect alleles. This method expedites the identification of climate-resilient grapevines by considering the cumulative effect of numerous small-effect loci. Yet, the application of genomic selection in grapevine breeding is still in its early stages and faces challenges due to the crop’s heterozygous genome and long generation times [130].

Transcriptomics, which examines the abundance and activity of transcripts in specific tissues or cells, is widely applied to identify genes involved in stress responses, such as high temperature [131]. Studies of transcriptomes provide a window into how transcript levels change under different stress conditions and over time. Tools such as RNA sequencing, microarrays, and expressed sequence tags are instrumental in understanding the stress-induced gene expression. Different grapevines exhibit varying levels of tolerance to stressors such as water deficiency, extreme temperatures, and diseases. For instance, some grapevine genotypes possess traits that enhance their ability to cope with water scarcity and different temperatures [71,132]. These traits include deeper root systems, efficient water use, and the ability to maintain cellular functions under stress [86]. High-throughput Illumina RNA sequencing (RNA-seq) has been widely used to profile transcriptomes in grapevine leaves [133]. High-throughput sequencing technologies enable researchers to identify specific genes associated with stress tolerance. For example, genes involved in the synthesis of protective compounds, such as antioxidants and osmoprotectants, are upregulated in response to stress [134].

In an integrative transcriptomic analysis [71], researchers uncovered a core set of stress-responsive genes, novel transcription factors, and regulatory elements that orchestrate the grapevine’s complex defense mechanisms governing stress tolerance by an intricate regulatory network involving coordinated changes in primary and secondary metabolism as well as hormone signaling pathways. Notably, the study identified key transcription factor families (such as *WRKY*, *MYB*, and *NAC*) as central hubs within these regulatory networks. Some of these transcription factors facilitate the rapid reprogramming of the transcriptome in response to environmental challenges, ensuring the timely activation of downstream protective responses. This discovery not only confirms the importance of previously known stress pathways but also reveals novel candidate genes that may serve as useful markers or targets in breeding programs aimed at enhancing grapevine resilience. Furthermore, these protective compounds play a vital role in mitigating the damaging effects of stress on grapevine cells.

Moreover, transcriptomic analyses reveal that grapevines activate a complex network of stress-responsive genes when exposed to adverse conditions. These genes are involved in various physiological processes, including photosynthesis, water transport, and cellular homeostasis [64]. Understanding the regulatory mechanisms that control the expression of these genes can provide valuable insights into the genetic basis of stress tolerance. For instance, ‘Montepulciano’ possesses traits that enable it to maintain high water use efficiency under water-deficient conditions [132,135]. Similarly, other grapevines, like ‘Cabernet Sauvignon’, ‘Chardonnay’, ‘Riesling’, and ‘Tocai Friulano’ exhibit little or no tolerance to very low temperatures [71,136]. Guo et al. [137] employed RNA-seq to quantify differential gene expression in ‘Kyoho’ grapevine leaves under heat stress, successfully identifying key transcription factors involved in photosynthesis regulation and abscisic acid signaling. Similarly, Tan et al. [138] integrated RNA-seq with gene co-expression network analysis using Weighted Gene Co-expression Network Analysis to dissect the regulatory networks activated in grapevine leaves during simultaneous water deficiency and heat stress conditions, thereby providing deeper insights into the plant’s adaptive mechanisms.

In addition to short-read sequencing, long-read transcriptome profiling techniques, such as PacBio Iso-Seq and Oxford Nanopore sequencing, have further enriched our understanding by capturing full-length transcripts and revealing complex alternative splicing events. For instance, Wang et al. [139] utilized both RNA-seq and full-length transcript sequencing to establish a genetic foundation for cold tolerance in grapevines. Their analysis not only pinpointed candidate genes and pathways involved in cold adaptation but also suggested that these genetic elements could serve as promising targets in breeding programs aimed at developing grapevines with improved stress tolerance. Jiang et al. [140] used high-depth RNA-seq along with splicing-aware algorithms to show that alternative splicing significantly enhances transcriptome and proteome diversity under heat stress, thereby contributing to improved adaptability. These alternative splicing events lead to the production of multiple protein isoforms from a single gene, which can be critical for stress adaptation.

### 4.4. Proteomics and Metabolomics

Proteomics has significantly advanced our understanding of grapevine physiology, particularly in response to environmental stresses. Early proteomic analyses, such as the study by Sarry et al. [141], identified 67 major proteins in ripe grape berries, providing insights into sugar and organic acid metabolism. Subsequent research expanded this knowledge. For instance, Giribaldi and Giuffrida [142] reviewed various proteomic approaches (from two-dimensional gel electrophoresis to more advanced methods like Isobaric Tag for Relative and Absolute Quantitation or Tandem Mass Tags) that enhance the identification and quantification of stress-responsive proteins in different grapevine tissues (berries, leaves, stems, roots, shoots, and cell cultures) under abiotic stresses. The application of proteomics helps in understanding grapevine responses to environmental challenges, including water deficiency and salinity stress [143]. Proteomic changes are often coupled with significant shifts in the metabolome, providing a more complete picture of the plant’s response.

Proteomics studies on grapevines under temperature stress identified key proteins involved in stress tolerance, such as heat shock proteins and cold-regulated proteins, which protect cells from temperature extremes [144]. Quantitative proteomics approaches, including isobaric tags for relative and absolute quantitation and label-free quantification techniques, reveal important proteins involved in temperature stress pathways. Proteins like dehydrins and Late Embryogenesis Abundant proteins are known to play significant roles in cold tolerance [145]. Comparative proteomics shows differential expression of proteins linked to cold adaptation [146]. Nonetheless, many stress-responsive proteins remain insufficiently annotated, limiting our understanding of their precise roles. Further research and validation of these proteins could enhance grapevine breeding for improved stress resilience [147].

Metabolomics approaches, including gas chromatography–mass spectrometry, liquid chromatography–mass spectrometry, and nuclear magnetic resonance spectroscopy, have been instrumental in profiling stress-induced metabolic changes [148]. These stress conditions significantly influence the metabolic profile of grapevines, revealing adaptive and stress-mitigation mechanisms. Temperature stress alters primary and secondary metabolites in grapevines. Under heat stress, specific sugars (e.g., glucose and fructose) and sugar alcohols (such as sorbitol) accumulate, likely aiding in osmotic regulation and protecting against cellular damage [149]. Moreover, heat stress induces changes in amino acid profiles, such as increased proline levels, which act as osmoprotectants and ROS scavengers [150]. Cold stress triggers an increase in metabolites associated with energy metabolism, such as organic acids (malate and citrate), as well as compounds involved in oxidative stress defense, including ascorbate and glutathione [151]. Secondary metabolites like flavonoids, anthocyanins, and stilbenes are also upregulated, contributing to antioxidant defense mechanisms and stabilizing cellular structures under low temperatures [152]. Water-deficit stress significantly affects the grapevine metabolome, with increased accumulation of polyphenols, including tannins and flavonoids, which enhance ROS scavenging and protect cells from oxidative damage. Osmolytes such as betaine and trehalose also accumulate, aiding in water retention and cell stability [153].

### 4.5. Integrative Omics and Meta-Analyses

Integrating transcriptomics with other omics approaches, as discussed by Xu et al. [133], has provided a comprehensive “omics” perspective on the plant’s response to environmental challenges. This multi-omics integration, often coupled with gene ontology and Kyoto Encyclopedia of Genes and Genomes pathway analyses, enables researchers to correlate transcriptional changes with shifts in metabolic pathways and protein function. Complementary techniques such as quantitative real-time PCR validate the expression levels of key candidate genes identified through high-throughput transcriptomic studies, thereby reinforcing the reliability of these findings.

Integration of metabolomics with transcriptomics and proteomics has provided deeper insights into the regulatory networks governing grapevine stress responses, offering potential targets for breeding stress-resilient grapevines [143]. For instance, Dal Santo et al. [154] demonstrated a coordinated interplay between primary metabolism and stress-responsive gene networks, like sugar metabolism, antioxidant defenses, and hormonal signaling pathways using omics analyses, further emphasizing the potential for targeted interventions to boost resilience. Moreover, emerging genomic resources, including high-quality reference genomes for grapevine, have enabled researchers to integrate multi-omics data to dissect stress-response pathways at an unprecedented resolution [45]. Savoi et al. [6] integrated transcriptomic and metabolomic resources of grapevine to study fruit metabolites. Their comprehensive work emphasizes the importance of combining omics data to understand the complex regulatory networks influencing grape berry composition. The research provides insights into the molecular mechanisms that determine fruit quality traits, such as flavor, color, and nutritional content. The authors identified key genes and metabolic pathways that, when targeted, can help breeding programs aimed at enhancing grapevine quality. The findings underscore the value of multi-omics strategies in improving grapevine tolerance to environmental challenges. These integrative approaches not only facilitate the identification of key regulatory genes and metabolic markers associated with stress tolerance but also support the development of molecular markers for breeding programs. To date, an increasing number of studies have conducted meta-analyses of transcriptomic datasets to investigate grapevine responses to water deficit stress [155]. These analyses have enabled the construction of gene co-expression networks associated with abiotic stress, facilitating the identification of key genes and regulatory pathways involved in stress adaptation. Such resources, including comprehensive transcriptomic atlases, provide valuable insights into the molecular mechanisms underpinning grapevine resilience to water-deficient conditions.

## 5. Epigenetic Regulation in Grapevine

Epigenetic mechanisms play a crucial role in the regulation of gene expression in response to environmental stressors. These mechanisms are sometimes referred to as an “epigenetic alphabet” [156]. In this concept, distinct epigenetic marks form a combinatorial code that modulates gene expression, including DNA methylation, histone modifications, and chromatin remodeling. Such modulation collectively contributes to the plant’s ability to adapt to environmental stresses. In grapevines, understanding these epigenetic processes is essential for developing strategies to enhance resilience and ensure sustainable viticulture. For example, gene *VvNCED1*, a key contributor to abscisic acid biosynthesis, exhibits altered promoter methylation under water-deficient conditions, which affects its expression and enhances stress adaptation [138]. Additionally, under heat stress, the gene *VvHsfA2,* encoding a heat shock transcription factor crucial for activating protective heat shock proteins, shows altered expression correlated with histone acetylation [138]. Similarly, the transcription factor *VvDREB2A*, a central player in mediating water-deficit responses, is regulated through histone modifications that fine-tune its activity during stress [157]. Moreover, the WRKY transcription factor *VvWRKY11* shows shifts in DNA methylation in response to water deficiency, influencing the regulation of downstream stress-responsive genes [157]. The expression of gene *VaCPK20*, encoding calcium-dependent protein kinase, is regulated through epigenetic modifications in response to water deficiency and cold stress [158].

### 5.1. Epigenetic Mechanisms in Plants

DNA methylation is one of the best-studied epigenetic modifications in plants. It involves the addition of a methyl group to the cytosine residues in DNA, which can lead to the repression of gene expression. In grapevines, DNA methylation plays a significant role in the response to both biotic and abiotic stresses [159]. For example, shifts in DNA methylation patterns in the promoter regions of defense-related genes, such as those regulating stilbene biosynthesis via transcription factors *VvMYB14* and *VvMYB15*, occur following pathogen attack. Similarly, the expression of *VvNAC17*, a transcription factor involved in tolerance to water deficiency, is modulated by DNA methylation changes under water deficit [160,161,162]. The maintenance methyltransferase *VvMET1* and de novo methyltransferase *VvDRM2* mediate responses to water deficiency and salinity, with reversible methylation changes observed at specific genomic regions [98,100], suggesting that vineyard management practices can influence these methylation profiles, thereby affecting both stress responses and fruit quality [157].

DNA methylation in plants can also happen via interaction with RNA, called RNA-directed DNA methylation (RdDM). RdDM plays a critical role in maintaining genome stability by silencing transposable elements and regulating gene expression, particularly in response to developmental and environmental signals [163]. RdDM is initiated by small interfering RNAs. RNA polymerase IV and RNA-dependent RNA polymerase 2 produce the precursors, while ARGONAUTE proteins bind the small interfering RNAs and direct them to complementary genomic sequences. Once targeted, the methylation of cytosine residues occurs across CG, CHG, and CHH contexts, facilitated by RNA polymerase V and other associated proteins. The ability of RdDM to modify gene activity adds another level of control in plant adaptation and stress resilience.

Histone modifications, including methylation, acetylation, and phosphorylation, are another key component of the epigenetic regulation of gene expression. These modifications can alter the structure of chromatin, making it more or less accessible to the transcriptional machinery. In grapevines, histone modifications are implicated in the response to various stresses. For example, histone methylation is linked to the regulation of genes involved in abiotic stress tolerance, such as those encoding for osmoprotectants and antioxidants [164]. On the other hand, histone acetylation is associated with the activation of defense-related genes in response to pathogen attack [165]. Berger et al. [166] emphasized that histone deacetylases play an indispensable role in fine-tuning these responses. Interestingly, several grapevine genes involved in histone deacetylation, such as *VvHDA6* encoding the histone deacetylase enzyme, were found to be upregulated in response to cold stress, contributing to enhanced freezing tolerance by fine-tuning the expression of cold-responsive genes [159,166]. The proper balance of acetylation and deacetylation is necessary for both activating stress-related genes and ensuring genomic stability under prolonged stress conditions. Mutations affecting these enzymes compromise stress tolerance, underscoring their potential as targets for boosting grapevine resilience.

Another mechanism epigenetically regulating gene expression is chromatin remodeling. Chromatin remodeling refers to the dynamic changes in the structure of chromatin. This process involves the repositioning or restructuring of nucleosomes, which can either facilitate or hinder the access of transcription factors to DNA. In grapevines, the SWI/SNF chromatin remodeling complex has a critical role in response to pathogens by facilitating the activation of defense-related genes through nucleosome repositioning [159,167,168].

### 5.2. Epigenetic Memory of Stress Responses

One of the fascinating aspects of epigenetic regulation is the concept of epigenetic memory, where plants “remember” past stress events and respond more effectively upon subsequent exposures. This “memory” enables grapevines to respond more effectively to recurrent stress events [169,170]. Detailed investigations in model plants revealed that dynamic changes in both DNA methylation and histone marks facilitate recovery after stress exposure. These reversible modifications allow for a rapid return to basal expression levels once the stress subsides, and prime the plant for future challenges. The memory can be transient or long lasting, and in some cases, it can be passed onto the next generation.

While robust evidence for stress memory and particularly transgenerational epigenetic inheritance in grapevines is still emerging [157,171,172], with much of the foundational research conducted in model plants, some studies in grapevines suggest the existence of epigenetic memory in response to biotic and abiotic stresses [172]. Plants experiencing pathogen attack can exhibit enhanced tolerance to subsequent infections, a phenomenon known as systemic acquired tolerance [161]. Similarly, grapevines exposed to water-deficit stress can develop a memory that allows them to respond more effectively to future water-deficit events [164,173].

Grapevine responses to water-deficit stress involve DNA methylation and chromatin remodeling of stress-responsive genes [160,161,173]. Such discoveries highlight the importance of studying epigenetic mechanisms of gene expression regulation in response to environmental stressors. Furthermore, it is hypothesized that epigenetic memory is not only critical for stress tolerance but may also influence terroir expression [174]. The concept posits that epigenetic marks, potentially shaped by local environmental conditions and viticulture practices, could influence the quality of wine, although this remains an active area of investigation.

Future research in epigenetic mechanisms involved in responses to environmental stress caused by climate change in grapevines should focus on the integration of multi-omics approaches ranging from genomics and proteomics to metabolomics and epigenetics with advanced CRISPR/Cas9 gene-editing technologies. This could provide valuable insights into the molecular basis of stress tolerance and support the development of stress-resistant grapevines without compromising fruit quality [175]. It will be crucial in such research to also evaluate potential pleiotropic effects, ensuring that enhancements in stress resilience do not inadvertently compromise fruit quality or other desirable traits.

## 6. Viticulture in a Changing Climate: Challenges and Opportunities

### 6.1. Environmental Challenges and Local Adaptation Strategies

Genetic diversity within grapevines is a critical component of their ability to withstand adverse conditions. Different grapevines exhibit varying levels of tolerance to stressors such as water deficiency, extreme temperatures, and diseases [1]. For instance, some genotypes possess traits that enable them to maintain cellular functions and water use efficiency under stress [132], while others may have deeper root systems that allow them to access water from deeper soil layers [176]. Recent advancements in genomics have facilitated the identification of specific genes associated with stress tolerance, providing valuable insights into the genetic basis of these traits [1,70]. Environmental factors also play a significant role in grapevine resilience. The interaction between grapevines and their environment is complex, with factors such as soil composition, water availability, and temperature influencing their growth and productivity. Climate change exacerbates these interactions, leading to shifts in phenological stages and potentially reducing the quality and quantity of grapevine yields. To mitigate these effects, researchers are exploring various adaptation strategies, including the selection of appropriate rootstocks [177], the implementation of efficient irrigation techniques [89,178], and the modification of vineyard management practices [119].

Climate change presents significant challenges for viticulture, influencing grapevine physiology, phenology, and, ultimately, wine quality. Droulia and Charalampopoulos [179] review the impacts of climate change on European viticulture, emphasizing regional conditions, potential shifts in suitable grape-growing areas, and the importance of selecting resilient grapevine varieties. In response to increasing water scarcity, Medrano et al. [180] discussed various irrigation management techniques that enhance water use efficiency, such as deficit irrigation, partial root-zone drying, and water reuse. Complementing these strategies, Gambetta et al. [181] provided an in-depth look at water-deficit stress physiology in grapevines and stress the need for breeding programs focused on tolerance to water deficiency along with sustainable water management practices. Meanwhile, Palliotti et al. [182] examined how canopy management practices can delay ripening under high temperature and water deficits, highlighting the significance of adjusting canopy structure to mitigate climate change effects on grapevine phenology. Finally, Van Leeuwen and Darriet [183] explore the effects of climate change on grape composition and wine quality, discussing how altering the grapevine microclimate through vineyard management, such as changing row orientation, adjusting plant density, and implementing shading as suggested by Hunter et al. [184], can help alleviate adverse impacts.

Collectively, environmental factors, including temperature, water availability, and soil conditions, significantly impact grapevine physiology and productivity. However, most studies focus on well-established viticulture regions in Europe and North America, while data from emerging or understudied regions, such as parts of Asia, South America, and Africa, remain sparse. Enhancing research efforts in these underrepresented regions is crucial for a truly global understanding of climate change impacts and for uncovering novel genetic resources for adaptation. Furthermore, the effects of different stressors on grapevine growth, yield, and quality vary significantly by region, yet comprehensive datasets comparing these variations are limited. Regional studies could provide valuable insights into how climate change is affecting global viticulture.

### 6.2. Future Perspectives

The challenges posed by climate change on viticulture are profound, demanding innovative strategies to enhance grapevine resilience. This short review examines how molecular approaches ranging from genomics through transcriptomics, proteomics, and metabolomics to epigenetics can improve our understanding of grapevine responses to stress. We also reflect on the importance of integrating these technologies into practical viticulture strategies, from breeding and vineyard management to sustainable production. Nevertheless, as a non-model woody perennial, grapevine research has not yet benefited from the fully optimized omics pipelines established for model species such as *Arabidopsis thaliana*, *Physcomitrium patens*, *Marchantia polymorpha*, *Nicotiana tabacum, Oryza sativa*, *Zea mays*, or even woody plant *Populus trichocarpa*, resulting in gaps in standardized workflows and comprehensive datasets.

The resilience of grapevines is inherently multifactorial, shaped by complex interactions between genotype, epigenetic regulation, and environment. Genetic diversity, especially within and among wild and indigenous grapevine taxa, remains a critical resource for future breeding programs. Expanding research to include these lesser-studied groups could increase the adaptive capacity of cultivated varieties in the face of accelerating climate variability. Extreme weather events are projected to become more frequent, directly threatening grapevine phenology and grape berry composition. While short-term mitigation strategies such as regulated deficit irrigation, canopy adjustment, or rootstock selection have demonstrated benefits in specific contexts, long-term adaptation requires deeper genetic insight [185]. Understanding how grapevines will evolve under prolonged climate shifts requires extended field trials and predictive modeling, which are currently insufficient. Despite promising advances in omics and gene-editing tools such as CRISPR/Cas9, their application in viticulture remains largely experimental. Stable field-level implementation is constrained by regulatory hurdles, transformation inefficiency, and public perception. Similarly, integrating multi-omics data to build predictive models of stress tolerance is still limited by a lack of long-term field validation and high-throughput phenotyping infrastructure.

Taken together, biodiversity, domestication, environmental stress, and epigenetic memory jointly underpin grapevine adaptation. Decoding the molecular interplay through integrative omics will be essential for designing effective breeding and management strategies. To ensure the long-term sustainability of viticulture, further investment in systems biology, expanded germplasm screening, and translational field research is required, bridging the gap between molecular discovery and real-world application.

## Figures and Tables

**Table 1:** Grapevine stress responses: impact, mechanisms, and key genes.

Stress Type	Affected Regions	Severity and Impact	Morphological Changes	Biochemical Responses	Molecular Mechanisms	Key Genes and Known Mechanisms
Heat stress	Mediterranean, California, Australia	Up to 30% yield loss in extreme heat years [84].	Accelerated phenology, smaller berries, leaf senescence, reduced shoot growth [85,86,87,88]	Increased abscisic acid, elevated flavonoid synthesis [89]	Activation of heat shock proteins and MAP kinase pathways counteract protein denaturation [90]	Heat Shock Proteins (HSP70, HSP90, HSF1, APX1), MAP kinase pathways [90]
Drought	Southern Europe, Chile, South Africa	20–40% reduction in grapevine vigor and berry size [91].	Reduced shoot growth, smaller berries [85,86,87,88]	Increased ABA induces stomatal closure [89]	ABA-responsive elements trigger drought-responsive genes [92]	*VvNCED1*, *VvDREB1/2*—ABA synthesis, *VvGRIK1, VvRFS2*, *VvLKR* drought-responsive genes [92,93,94]
Cold stress	Canada, Eastern Europe	50–100% bud mortality during severe frost events [95]	Bud necrosis, shoot dieback [95,96]	Accumulated proline and soluble sugars protecting cells against freezing [97]	ICE1-CBF-COR cascade regulates cold-responsive genes, enhancing freezing tolerance [98]	*VvCBF4*, *VvICE1*—ICE1-CBF-COR cascade, enhancing freezing tolerance [98]
UV radiation	Arid regions, coastal vineyards	Impaired photosynthesis, enhanced flavonoid synthesis [81]	Leaf bronzing, cuticle thickening [81]	Enhanced production of UV-absorbing compounds like anthocyanins and flavonols [81]	ROS scavenging pathways, involving superoxide dismutase and catalase, mitigate oxidative damage [81]	*VvMYB4*—UV shielding and antioxidant defenses [81]
Soil salinity	Coastal vineyards, irrigated areas	Disrupted ion homeostasis, osmotic stress [99]	Reduced growth, leaf chlorosis [100]	Increased ABA for stomatal regulation [101]	Ion homeostasis mechanisms reduce salt toxicity [100]	*VvNHX1*—Ion transport, maintaining osmotic balance [102]
Spring frost	Northern Europe, Canada, USA	Severe bud damage and yield loss in early-budding grapevines [78]	Bud necrosis, shoot dieback [79]	Increased soluble sugars to lower freezing point [79]	ICE1-CBF-COR pathway activation protects cellular structures [98]	*VvCBF2*, *VvCBF3*—Frost tolerance via cold-responsive gene activation [98]
Water deficit	Mediterranean, California, Australia	Reduction in yield by 20–50%, smaller berries, reduced canopy growth [103]	Decreased leaf area, reduced shoot growth, and smaller berries [85,86,87,88]	Increased ABA, enhanced flavonoid synthesis [91]	Upregulation of drought-responsive genes, enhanced expression of dehydration-responsive elements [92]	*VvNCED1*, *VvDREB2*—ABA synthesis, drought-responsive genes [92]

## Abbreviations

The following abbreviations are used in this manuscript:

ABA	Abscisic Acid
Cas9	CRISPR-associated protein 9
CBF	C-repeat Binding Factor
COR	Cold-Responsive gene
CRISPR	Clustered Regularly Interspaced Short Palindromic Repeats
GWASs	Genome-Wide Association Studies
HSP	Heat Shock Protein
ICE1	Inducer of CBF Expression 1
KEGG	Kyoto Encyclopedia of Genes and Genomes
RdDM	RNA-directed DNA Methylation
RNA-seq	RNA sequencing
ROS	Reactive Oxygen Species
SNP	Single-Nucleotide Polymorphism
TFs	Transcription Factors
